# Natural microbial polysaccharides as effective factors for modification of the catalytic properties of fungal cellobiose dehydrogenase

**DOI:** 10.1007/s00203-021-02424-1

**Published:** 2021-06-16

**Authors:** Justyna Sulej, Magdalena Jaszek, Monika Osińska-Jaroszuk, Anna Matuszewska, Renata Bancerz, Monika Janczarek

**Affiliations:** 1 Department of Biochemistry and Biotechnology, Institute of Biological Sciences, Maria Curie-Sklodowska University, Akademicka 19, 20-033 Lublin, Poland; 2 Department of Genetics and Microbiology, Institute of Biological Sciences, Maria Curie-Sklodowska University, Lublin, Poland

**Keywords:** Polysaccharides, Cellobiose dehydrogenase, Scavenging effect, Fungi

## Abstract

Polysaccharides are biopolymers composed of simple sugars like glucose, galactose, mannose, fructose, etc. The major natural sources for the production of polysaccharides include plants and microorganisms. In the present work, four bacterial and two fungal polysaccharides (PS or EPS) were used for the modification and preservation of *Pycnoporus sanguineus* cellobiose dehydrogenase (CDH) activity. It was found that the presence of polysaccharide preparations clearly enhanced the stability of cellobiose dehydrogenase compared to the control value (4 °C). The highest stabilization effect was observed for CDH modified with Rh110EPS. Changes in the optimum pH in the samples of CDH incubated with the chosen polysaccharide modifiers were evidenced as well. The most significant effect was observed for Rh24EPS and Cu139PS (pH 3.5). Cyclic voltammetry used for the analysis of electrochemical parameters of modified CDH showed the highest peak values after 30 days of incubation with polysaccharides at 4 °C. In summary, natural polysaccharides seem to be an effective biotechnological tool for the modification of CDH activity to increase the possibilities of its practical applications in many fields of industry.

## Introduction

The development of methodologies to stabilize the structure of enzymes to exploit their full potential as catalysts, in particular molecules with multiple subunits and cofactors, seems to be very important for application in industry. A few factors are known to promote changes in the spatial configuration and thus the loss of catalytic activity and stability of an enzyme, viz. temperature, pH, chemical agents, autolysis (proteases), or ionic strength (Iyer and Ananthanarayan 2008). Inactivation may occur by dissociation of subunits of multimeric enzymes or their cofactors, denaturation of tertiary or secondary structures as well as aggregation, coagulation or chemical decomposition of molecules (Lencki et al. 1992). There are many different methods for the stabilization of enzymes with respect to their use in various environmental conditions. One of the popular strategies used by living organisms to increase protein stability is the employment of osmolytes like sugars, polyols, or neutral amino acids. These molecules are able to stabilize protein structure towards changing the solvent properties of the surrounding water and thus the interaction between proteins and solvents (Taravati et al. 2007). The protective effect of disaccharide on proteins is often greater than that of other agents. Carbohydrate osmolytes, such as sucrose or trehalose, are also known to protect biological molecules against loss of their catalytic activity or chemical and thermal denaturation (Kaushik and Bhat 2003). Hence, disaccharides are often used in the freeze-drying process of aqueous solutions of proteins as the most effective cryo- and lyoprotective excipients (Timasheff 1998). Recently, polysaccharides have also been proposed as promising biomolecules used for maintenance of the biocatalytic functions of an enzyme, including its activity and stability. This group of biological polymers is found in almost all living organisms such as seaweeds (alginate, agar–agar, and carrageenan), plants (cellulose, hemicelluloses, pectin, and guar gum), microorganisms (dextran and xanthan gum), and animals (hyaluronan, chondroitin, chitin, and heparin) (Karaki et al. 2016). The simple protein–polysaccharide interaction is an efficient technique to enhance the stability of enzymes. Polysaccharides can interact with biocatalysts by either covalent or non-covalent binding (Jadhav et al. 2014). The influence of polysaccharides on enzymatic activity and stability is probably related to two aspects. On the one hand, the interaction between polysaccharides and enzymes results in some conformational changes in enzyme molecules. On the other hand, the electrostatic interaction between a polysaccharide and a substrate was another factor influencing enzyme activity by affecting the enzyme and substrate combination (Li et al. 2010).

Cellobiose dehydrogenase (CDH, EC 1.1.99.18) is a well-known enzyme that has been intensively investigated in the last decade for application in many fields such as lignocellulose hydrolysis by acting as an electron source for lytic polysaccharide monooxygenases (LPMOs) (Kracher et al. 2016), biosensors (Stoica et al. 2006), biofuel cells (Harreither et al. 2012), bioremediation (Wingate et al. 2005), textile bleaching purposes (Flitsch et al. 2013), and recently for clinical applications (Nyanhongo et al. 2017). CDH is an extracellular glycoprotein secreted by many species of wood-decaying fungi (Ludwig et al. 2010). It is usually a monomeric protein consisting of two domains connected by a proteolytically sensitive linker region comprising approximately 20–35 amino acid residues. In vitro, the linker can be cleaved via proteolytic digestion resulting in a separate but still catalytically active flavin domain (DHcdh) with a flavin adenosine dinucleotide cofactor and a cytochrome domain (CYTcdh) with a b-type heme cofactor (Henriksson et al. 1991). DHcdh is the catalytically active domain of the enzyme and a member of the glucose-methanol-choline oxidoreductase (GMC) family, whereas the smaller CYTcdh acts as a built-in mediator (Ludwig et al. 2013). β-1,4-linked di- and oligosaccharide breakdown products of cellulose such as cellobiose or cellodextrins are the natural substrates for the catalytic activity of CDHs. Also, lactose is readily converted by CDHs, due to its very similar structure, although it is certainly not a natural substrate. The catalytic mechanism of CDH comprises both the oxidative and reductive half-reaction. For example, the cellobiose substrate is oxidized to cellobiono-1,5-lactone, which is spontaneously hydrolyses to cellobionic acid (Zamocky et al. 2006). The oxidative half-reaction can involve one- or two-electron acceptors which are directly reduced by the FADH_2_ or consecutive intramolecular single electron transfer from FADH_2_ to the heme cofactor and further to an external single electron acceptor like LPMO, cytochrome c, or an electrode surface (Kracher and Ludwig 2016; Scheiblbrandner and Ludwig 2019). Recently, increasing interest in CDH in terms of industry, life sciences and biomedical research as an antimicrobial and antibiofilm agent has been observed (Bollella et al. 2017; Henriksson et al. 2000; Ludwig et al. 2013; Nyanhongo et al. 2017). Application of CDH in all proposed areas requires high stability of the enzyme and, in particular, maintenance of its intact form with a cytochrome domain. A review of previous literature focused on the stabilization of CDH shows only reports of the use of sugars as effective protective substances in the freeze-drying process (Fischer et al. 2014) and stabilization of the enzyme with genetic engineering techniques (Balaž et al. 2019). There are no available research regarding the possibilities of the use of naturally produced fungal and bacterial EPSs as protective agents and modulators of CDH catalytic activity.

The main objective of the present work was to compare the catalytic properties of *P. sanguineus* CDH in incubation with bacterial exopolysaccharides extracted from *Rhizobium leguminosarum bv. trifolii* Rt24.2 (Rh24EPS), *Bradyrhizobium elkanii* USDA76 (Rh76EPS), *Bradyrhizobium japonicum* USDA110 (Rh110EPS), *Sinorhizobium meliloti* Rm1021 (Rh1021EPS), and fungal polysaccharides from *Cerrena unicolor* (Cu139PS) and *Ganoderma applanatum* (Ga261EPS). The analysis was focused on the investigation of the activity and storage stability of the enzyme, changes in optimum pH and antioxidant properties, as well as kinetic and electrochemical parameters. To the best of our knowledge, the work presented here is the first study of the modification of CDH functional properties by polysaccharides proposed in the literature.

## Materials and methods

### Microorganisms and culture conditions

The fungal strain *Pycnoporus sanguineus* (FCL199) was obtained from the culture collection of the Agriculture University, Tokyo, Japan (FCTUA) and deposited in the Fungal Collection (FCL) of the Department of Biochemistry and Biotechnology, Maria Curie-Sklodowska University, Lublin, Poland (ITS sequence deposited in GenBank under accession number JF308951). The culture was maintained on 3% (w/v) malt extract agar plates at 25 °C and stored at 4 °C. The inoculum was prepared by taking pieces of agar plates with the growing mycelium and transferring them to conical flasks containing Lindenberg and Holm medium (Lindeberg and Holm 1952) and incubated for 10 days at 25 °C. After 10 days of growth in stationary culture, the mycelium was homogenized in a disperser homogenizer T18 basic ULTRA-TURRAX (IKA, Staufen, Germany). The fragmented mycelial culture (10%, v/v) was used as a standard inoculum for further analysis. For cellobiose dehydrogenase production, the fragmented mycelium of the *P. sanguineus* strain was grown on a cellulose-based medium (Fang et al. 1999). The growth medium had the following composition (per litre): 2 g Avicel, 10 g (NH_4_)_2_HPO_4_, 1 g KH_2_PO_4_, 0.3 g MgSO_4_·7H_2_O_2_, 0.08 g CaCl_2_, 5 mg ZnSO_4_·7H_2_O, 1.5 mg MnSO_4_·4H_2_O,

1.5 mg CoCl_2_·6H_2_O, 5 mg FeSO_4_·7H_2_O, 100 mg yeast extract, and 0.1 mg thiamine. The pH of the medium was adjusted to 4.0 with 5 M HCl. After inoculation, the cultures were incubated at 28 °C on an incubator shaker Multitron (Infors, Bottmingen, Switzerland) at 120 rpm for 10 days. Bacterial strains *R. leguminosarum bv. trifolii* Rt24.2 (Janczarek and Skorupska 2007), *B. elkanii* USDA76 (Kuykendall et al. 1992), *B. japonicum* USDA110 (Kaneko et al. 2002), and *S. meliloti* Rm1021 (Meade and Signer 1977) were used for preparation of bacterial EPSs (Rh24EPS, Rh76EPS, Rh110EPS, and Rh1021EPS). The bacterial strains were grown on 79CA medium, pH 7.2 (Vincent 1970), supplemented with 1% glycerol and 0.1% succinate as a carbon source. The bacterial cultures were maintained in a 1-L flask with 500 mL of 79CA medium and incubated at 28 °C on an incubator shaker at 120 rpm for 4 days until the optical density of the cultures at 600 nm was 0.9. The bacteria were separated from the culture liquid by double centrifugation at 7500×*g* for 30 min. at 4 °C. The supernatants were used for the preparation of bacterial EPSs as described in our previous papers (Osińska-Jaroszuk et al. 2018).

The fungal EPSs (Ga261EPS) were isolated from *Ganoderma applanatum* (FCL261) deposited in the Fungal Collection (FCL) of the Department of Biochemistry and Biotechnology Maria Curie-Sklodowska University, Lublin, Poland. The conditions of the culture of the *G. applanatum* strain used in this paper were described in our previous work (Osińska-Jaroszuk et al. 2014a). In turn, fungal strain *Cerrena unicolor* 139 (Bull. ex Fr.) Murr. was obtained from the Regensburg University culture collection, deposited in the fungal collection (FCL) of the Department of Biochemistry and Biotechnology, Maria Curie-Sklodowska University, Lublin, Poland (ITS sequence deposited in GenBank under accession number DQ056858), and used to obtain fungal PSs (Cu139PS). The culture conditions and biomass preparation used for the isolation of crude polysaccharides were described in our previous work (Jaszek et al. 2013).

### Extraction of polysaccharides (PSs)

Crude exopolysaccharides were obtained according to procedures described previously (Bancerz et al. 2016; Osińska-Jaroszuk et al. 2018). The exopolysaccharides from bacterial and fungal culture liquid were precipitated with cold 96% ethanol in the ratio of 1:4 (v/v) and stored overnight at 4 °C. The precipitated exopolysaccharides were collected by centrifugation (10000×*g*, 30 min), dried at 20 °C to remove residual ethanol, dissolved in distilled water, and lyophilized. Before use, the freeze-dried preparations were dissolved with 10 mL of distilled water to a final concentration of 1 mg/mL. The crude polysaccharides obtained from *C. unicolor* were prepared according to the method described in our previous work (Osińska-Jaroszuk et al. 2017). The polysaccharides were extracted from the dried mycelia with hot water (90 °C, 4 h) in a 1:100 (w/w) ratio, cooled, and centrifuged at 9.000×*g* for 20 min. Next, the crude polysaccharide was precipitated from the supernatant by four volumes of cold 96% ethanol and stored overnight at 4 °C. The precipitated polysaccharides were collected by centrifugation (9000×*g*, 20 min) and washed three times with ethanol. Next, the samples of the polysaccharide were dissolved in distilled water (1 mg/mL) and used for further testing. Table 1 shows the characteristics of the polysaccharides used in this study.

**Table 1:** Characterization of polysaccharides

Strain		*G. applantum*		*C. unicolor*		*R. leguminosarum* bv. *trifolii* Rt24.2		*B. elkanii* USDA76		*B. japonicum* USDA110	*S. meliloti* Rm1021	
Source of polysaccharides		Fungi/mycelium		Fungi/mycelium		Bacteria		Bacteria		Bacteria	Bacteria	
Type of polysaccharides		Exopolysaccharide β-glucan		Endoplysaccharide (1 → 3)-α-d-glucan		Exopolysaccharide octasaccharide units		Exopolysaccharide tetrasaccharide units		Exopolysaccharide pentasaccharide units	Exopolysaccharide EPS I—succinoglycan EPS II—galactoglucan	
Composition of monosaccharides		Glucose, mannose		Glucose, xylose, mannose		d-glucose, d-glucuronic acid and d-galactose (5:2:1)		L-rhamnose and 4-*O*-methyl-d-glucuronic acid (3:1)		d-mannose, d-galacturonic acid, d-glucose and d-galactose (1:1:2:1)	EPS I- seven residues of d-glucose and one residue of d-galactose; EPS II—d-glucose and d-galactose (1:1)	
Linkage type		**–**		α-1,3,α-1,4 and α-1,3,4		β-1,3 and β-1,4		β-1,3, β-1,4 and α-1,4		β-1,3, β-1,4 and α-1,3	EPS I- β-1,3, β-1,4 and β-1,6; EPS II—α-1,3 and β-1,3	
Solubility		Water (60 °C)		1 M NaOH		Water		Water		Water	Water	
pH		**–**		**–**		6.6		8.0		7.3	7.5	
References		Osińska-Jaroszuk et al. (2014a, b)		Osińska-Jaroszuk et al. (2017)		Janczarek (2011) and Osińska-Jaroszuk et al. (2018)		Janczarek (2011) and Osińska-Jaroszuk et al. (2018)		Janczarek (2011) and Osińska-Jaroszuk et al. (2018)	Janczarek (2011) and Osińska-Jaroszuk et al. (2018)	

### Cellobiose dehydrogenase isolation and purification

*Pycnoporus sanguineus* CDH was isolated and purified according to the method described in our previous work with slight modification (Sulej et al. 2013). The culture fluid obtained after mycelium separation was centrifuged at 4 °C for 30 min at 12,000×*g* on a K615 centrifuge (Sigma, USA). The clear supernatant was concentrated on the Pellicon 2 Mini holder (Merk-Millipore, Germany) with an Ultracell mini cartridge (10 kD cut-off). The concentrated CDH solution was precipitated by ammonium sulphate between 40 and 90% saturation and the precipitate was collected by centrifugation (10,000×*g* for 30 min). The precipitate was dissolved in distilled water and desalted by diafiltration through centrifugal concentrators (Vivaspin Turbo 15) in polyethersulfone (PES) with a cut-off of 10 kDa (Sartorius, Göttingen, Germany). The diafiltrated sample was fractionated on a DEAE-Sepharose Fast Flow column (5 × 15 cm, GE Healthcare, Uppsala, Sweden) equilibrated with 50 mM sodium acetate buffer (pH 5.0). Elution was performed at a flow rate of 3 mL/min with the same acetate buffer. Bound proteins were eluted using a linear gradient from 0 to 100% of 0.5 M NaCl. Fractions containing cellobiose dehydrogenase activity (intact protein with flavin and heme domains) were pooled out and concentrated using a 30 kDa cut-off PES Vivaspin Turbo 15 (Sartorius, Göttingen, Germany). The purified proteins were stored at − 20 °C until further use.

### Determination of CDH activity and catalytic constants

Cellobiose dehydrogenase activity was determined for lactose oxidation measured at 30 °C in 100 mM sodium acetate buffer at pH 4.5 using 2,6-dichloroindophenol (DCIP) (Sigma Chemical Co., St. Louis, MO, USA) as electron acceptor according to the method proposed by Baminger et al. (Baminger et al. 2001) with modifications (Sulej et al. 2015). The analysis was conducted in 48 well microplates using a Tecan Infinite M200 Pro (Tecan, Zürich, Switzerland) plate reader. The assay mixture (1 mL) contained 100 µL of a properly diluted CDH sample, 100 µL of lactose (300 mM in 100 mM sodium acetate buffer, pH 4.5), and an appropriate amount of the same buffer. The mixture was incubated at 30 °C for 10 min. After that time the reaction was initiated by the addition of 50 µL of 3 mM DCIP (solution in water containing 10%, v/v ethanol) and the decrease in absorbance was monitored during the first 60 s. One unit of the enzyme activity (U) was defined as the amount of the enzyme reducing 1 µmol DCIP per minute in the described assay conditions. This assay was used for the determination of the activity of the native enzyme and the catalytically active flavin domain. Alternatively, the CDH activity of the intact protein containing both the flavin and heme domains was determined by monitoring the reduction of cytochrome c (20 µM) at 30 °C (Sigma Chemical Co., St. Louis, MO, USA) in 100 mM sodium acetate buffer, pH 4.5 (Sigma Chemical Co., St. Louis, MO, USA) in the presence of lactose (30 mM). The extinction coefficient (*ε* 550) was 19.6/mM/cm (Canevascini et al. 1991). The increase in absorbance at 550 nm after 10-min incubation at 30 °C was associated with the oxidation of the substrate (lactose) and measured on a microplate reader (Tecan Infinite 200 Pro). One unit of CDH activity was defined as the amount of enzyme that reduces cytochrome c at a rate of 1 µmol/min in the selected assay conditions (pH 4.5, 30 °C). The protein concentration was determined using the Bradford method (Bradford 1976) with crystalline bovine serum albumin (BSA) as a standard. All the measurements were taken in triplicate, and the results were recorded as the mean ± standard deviation of the means. The kinetic parameters (*K*_m_, *V*_max_, and *k*_cat_) of CDH in the presence of exopolysaccharides in comparison to the control were determined by direct regression of the Michaelis–Menten hyperbola obtained experimentally. The assays were conducted using a CDH sample and two different substrates (cellobiose and lactose) with DCIP as an electron acceptor. The concentration of cellobiose ranged from 0.05 to 1 mM, whereas the concentration of lactose ranged from 0.5 to 100 mM. Triplicates were run to ensure reliable determination of the kinetic parameters.

Catalytic constants were calculated using nonlinear curve fitting according to the Michaelis–Menten equation:

$$V = \left( {V_{{\max }} C} \right)/\left( {K_{{\text{m}}} + C} \right),$$

where *V* is the CDH activity and *C* is the cellobiose or lactose concentration. The data analysis was performed with the OriginPro8 software (OriginLab Corporation, Northampton, MA, USA).

### Effect of the PS from bacteria and fungi on the CDH activity and stability

The effect of PS on CDH activity was determined by measuring enzyme activity in the presence of polysaccharides isolated from the following bacterial strains: *R. leguminosarum bv. trifolii* Rt24.2 (Rh24EPS), *B. elkanii* USDA76 (Rh74EPS), *B. japonicum* USDA110 (Rh110EPS), and *S. meliloti* Rm1021 (Rh1021EPS) and fungal strains (*G. applanatum* (Ga261EPS) and *C. unicolor* (Cu139PS). CDH samples containing distilled water instead of polysaccharides (PS) were the control. The samples were prepared in conical 15-mL tubes by mixing the enzyme solutions with equal volumes of polysaccharides in a ratio of 1:1 (at the final concentration of 0.05%). The samples were incubated at 4 and 25 °C for 15, 30, and 60 days. CDH activity was assayed using the standard procedures described above with lactose as a reaction substrate and DCIP or cytochrome c as an electron acceptor. Simultaneously, the effect of pH on the activity of CDH in the presence of PS was analyzed in a pH range of 3.0–6.5 using 0.1 M citrate–phosphate buffer. The activity was determined in standard conditions with lactose and DCIP.

### Electrophoretic detection of CDH activities and protein visualization

Native polyacrylamide gel electrophoresis (PAGE) was applied for detection of the relative CDH activity. Electrophoretic PAGE analysis was performed using 10% polyacrylamide gel and 0.025 M Tris–glycine, pH 8.3 electrode buffer prepared according to the Laemmli procedure (Laemmli 1970). The analysis was conducted on a Bio-Rad Mini Protean system at a constant voltage of 150 V. After electrophoretic separation, the gels were washed with deionized water and then immersed in a 2 mM DCIP solution in 10% ethanol for 15 min. The detection of CDH activity bands was carried out with the addition of 1 mL of a 300 mM lactose solution (Sigma Chemical Co., St. Luis, USA) at 25 °C. Stained gels were scanned using a G:Box apparatus (Syngene, USA). Gel images were acquired by the G:Box system (Syngene, USA).

### Antioxidant activity determined by the DPPH radical scavenging assay

The antioxidant properties of CDH in the presence of PSs from the studied microorganisms was investigated using the DPPH assay according to an adapted spectrophotometry procedure described by Paduch et al. (2008) with slight modifications. Lactose or cellobiose was added to the reaction mixture as substrates of the enzyme, as described previously (Sulej et al. 2013, 2015). Standards (Trolox and Vitamin C) that are well known for their strong antioxidant activity were used as a positive control. The DPPH (2,2-diphenyl-1-picryl-hydrazyl-hydrate) free radical method is based on electron-transfer. The stable free radical (DPPH solution in ethanol) with violet color reacts with an antioxidant compound, which can donate hydrogen, and is reduced and discolored. The absorbance was measured spectrophotometrically at 515 nm using a microplate reader (Tecan Infinite 200 Pro) after 15 min of incubation at room temperature. All measurements were performed in triplicate.

The percentage of reduction of the DPPH oxidation rate was calculated according to the following formula:

$${\text{DPPH scavenging effect}}\left( \% \right) = \left[ {\left( {A0 - A1} \right)/A0} \right] \times 100$$

where *A*0 means the absorbance of the control sample and *A*1 means the absorbance of the standards or tested compounds.

### Electrochemical experiments

All electrochemical measurements were performed on the Eco Chemie Autolab potentiostat with GPES software (GPES, version 4.9, Eco Chemie, the Netherlands) with a three-electrode configuration. The three-electrode electrochemical cell contained a standard Ag/AgCl (1 M KCl) electrode, a platinum wire as a counter electrode, and a glassy carbon electrode (GCE, BAS) as a working electrode. Before each experiment, the GCE electrode was polished with aluminum oxide powder (grain size down to 0.05 m) on a wet pad, rinsed with water and ethanol, and dried at room temperature. Cyclic voltammetry measurements of CDH samples with polysaccharide were conducted in a 5-mL vessel at ambient temperature (25 °C) using an acetic buffer (50 mM, pH 5.0) as a supporting electrolyte, 30 mM lactose as a substrate, and 0.2 mM [K_3_Fe(CN)_6_] as an electron transfer mediator. The studies were carried out at the potential of − 200 + 800 mV at a rate of a potential change of 1 mV/s.

### Statistical analysis

The results were expressed as the mean ± SD of three independent experiments (*n* = 3). The mean values and standard deviation were calculated using the Excel program (Microsoft Office 2010 package). Values of *p* ≤ 0.05 were considered as statistically significant.

## Results and discussion

### Effect of bacterial and fungal exopolysaccharides on cellobiose dehydrogenase biochemical properties

#### Enzyme activity and storage stability

**Fig. 1:**
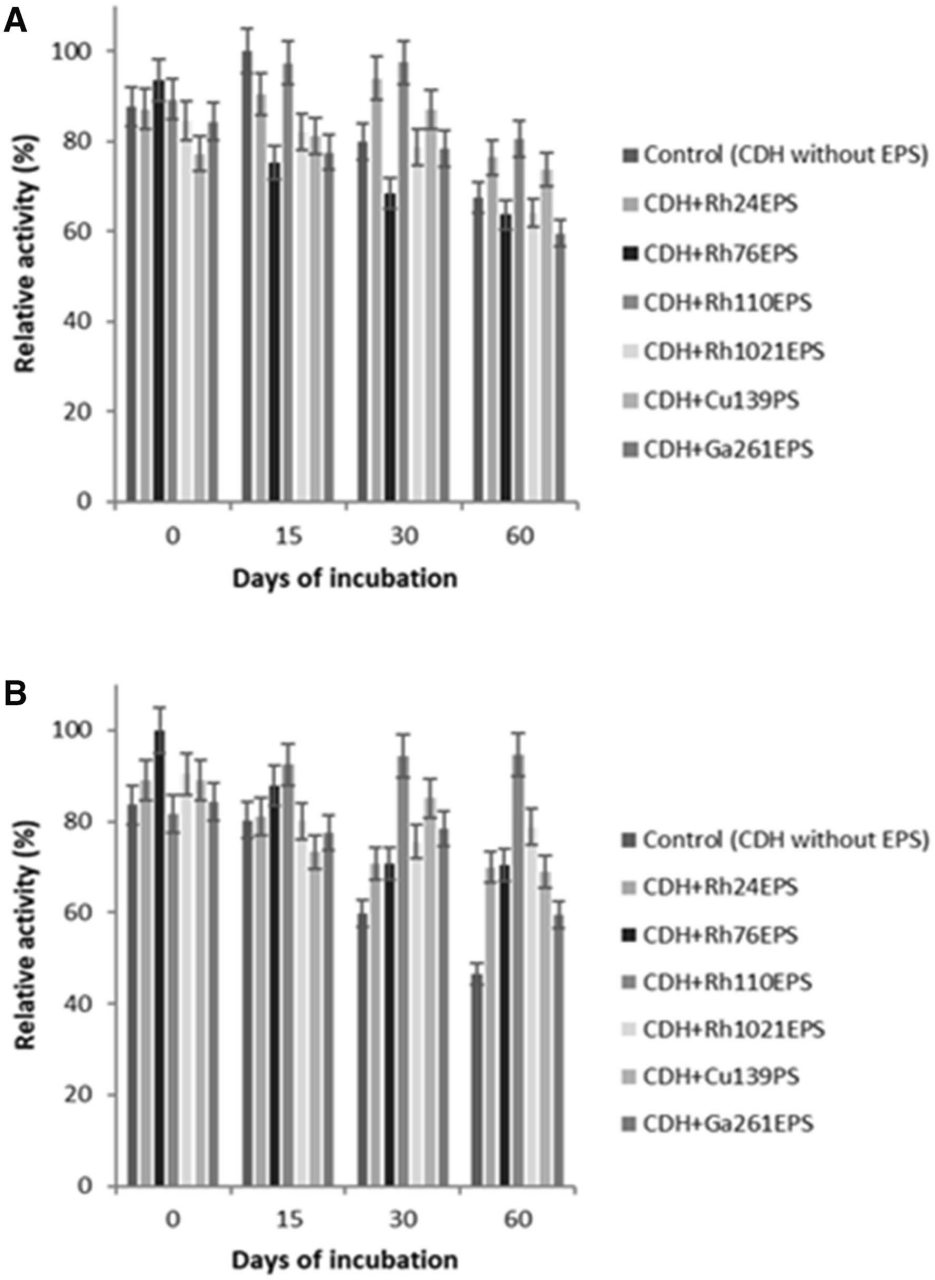
Effect of the incubation time on cellobiose dehydrogenase activity and stability in the presence of bacterial and fungal polysaccharides. **A** DCIP and **B** cyt c as electron acceptors. The experiments were performed in triplicate and relative activity was calculated from the activity at 4 °C and time of incubation 0, 15, 30 and 60 days. The average relative value (*n* = 3) and error bars are shown. Values with different letters are significantly different (*p* ≤ 0.05)

In the present study, the effect of bacterial and fungal polysaccharides on *P. sanguineus* cellobiose dehydrogenase activity and storage stability was determined for the first time. Samples of fungal CDH were incubated in the presence or absence of RhEPS, CuPS, and GaEPS preparations for 0, 15, 30, and 60 days at 4 °C. The activity was measured with the use of two activity assays: the DCIP assay (unspecific detection of both heme-containing untruncated CDH and the single flavin domain) and the cytochrome c assay (specific for intact enzyme molecules). Incubation of the enzyme with RhEPS, CuPS, and GaEPS showed an influence of both fungal and bacterial PSs on the stability and intact structure of CDH, compared to the control values (without PS addition). Significantly reduced CDH activity measured with cytochrome c (only 46% of retained basal activity) was observed after 60-day storage of the enzyme without polysaccharide probes at 4 °C. All the polysaccharides used were able to enhance the stability of cellobiose dehydrogenase. The highest stabilization effect was detected in the case of CDH modified with Rh110EPS (Fig. 1). It is evident that CDH with Rh110EPS had higher residual activity than free CDH at any point of time.

The stabilizing effect of polysaccharides has been reported for some other industrially relevant enzymes such as lipase (Bancerz et al. 2016), laccase (Jadhav and Singhal 2013; Osińska-Jaroszuk et al. 2018), alcohol dehydrogenase (Jadhav et al. 2014), or α-amylase (Jadhav and Singhal 2014). A similar level of storage stability of glucose oxidase (Altikatoglu and Basaran-Elalmis 2012) and horseradish peroxidase (Altikatoglu and Basaran 2011) in similar conditions has been obtained using dextran as a protective polysaccharide. Another effect of polysaccharides on the stability of the same enzyme results from the differences in their structure and chemical nature. They can bind to the same enzyme in a unique and specific way thus attributing different functional properties to the respective enzyme (Kagliwal and Singhal 2014). Stabilization of the native structure of the enzyme at almost the same level for more than 8 weeks seems to be extremely important in bioelectrochemical applications e.g. biosensors and biofuel cells. Efficient direct electron transfer (DET) in CDH relies on good communication between the cytochrome domain (CYT) and the electrode. Therefore, it is relevant to maintain the binary structure of the enzyme (Bollella et al. 2018). The effective stabilization of CDH only with sugars as cryoprotectants during freezing and freeze-drying (Fischer et al. 2014) has been investigated, but no studies are available on the CDH storage protection effect with polysaccharides.

#### Effect of pH on enzyme activity and storage stability

**Fig. 2:**
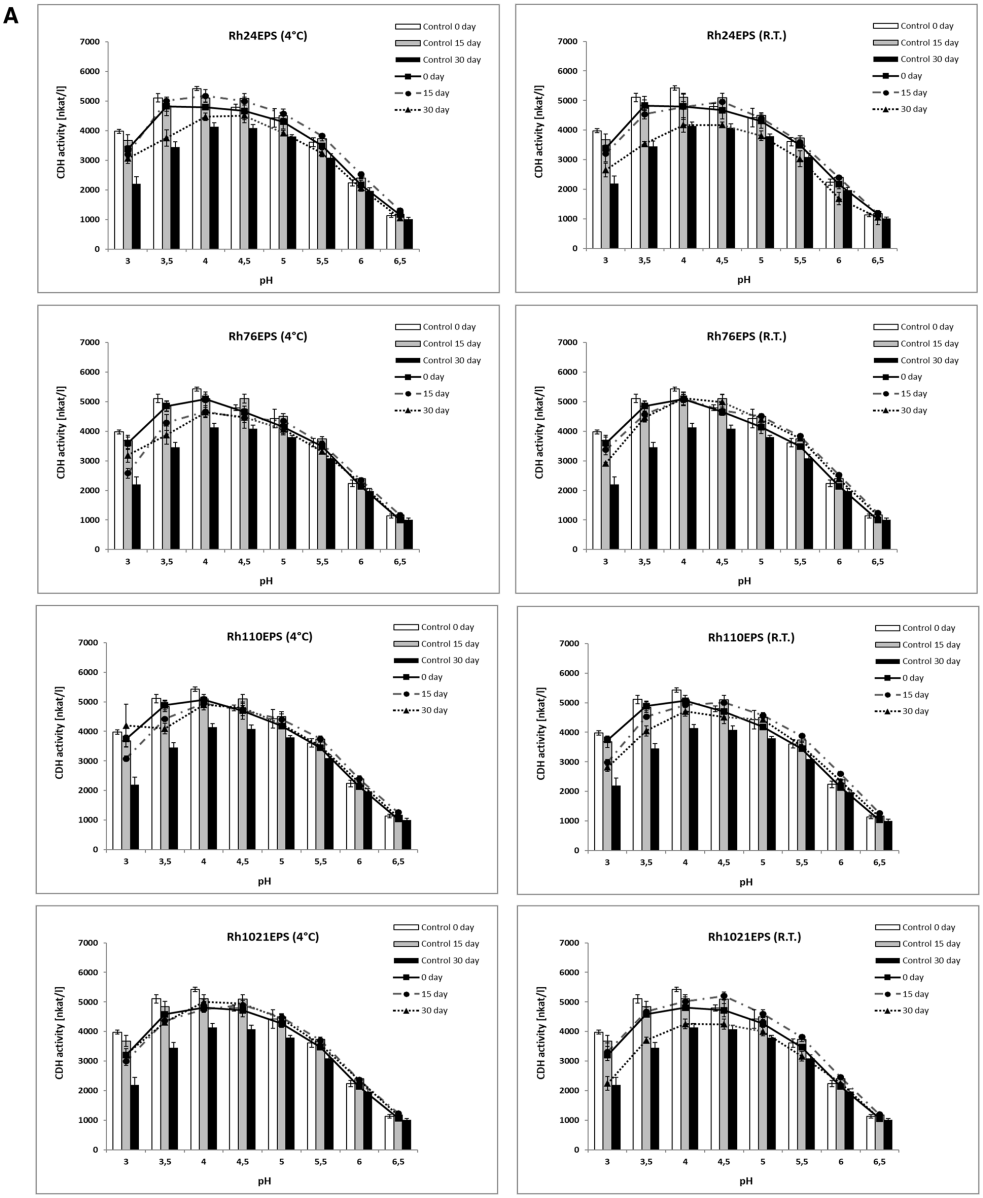

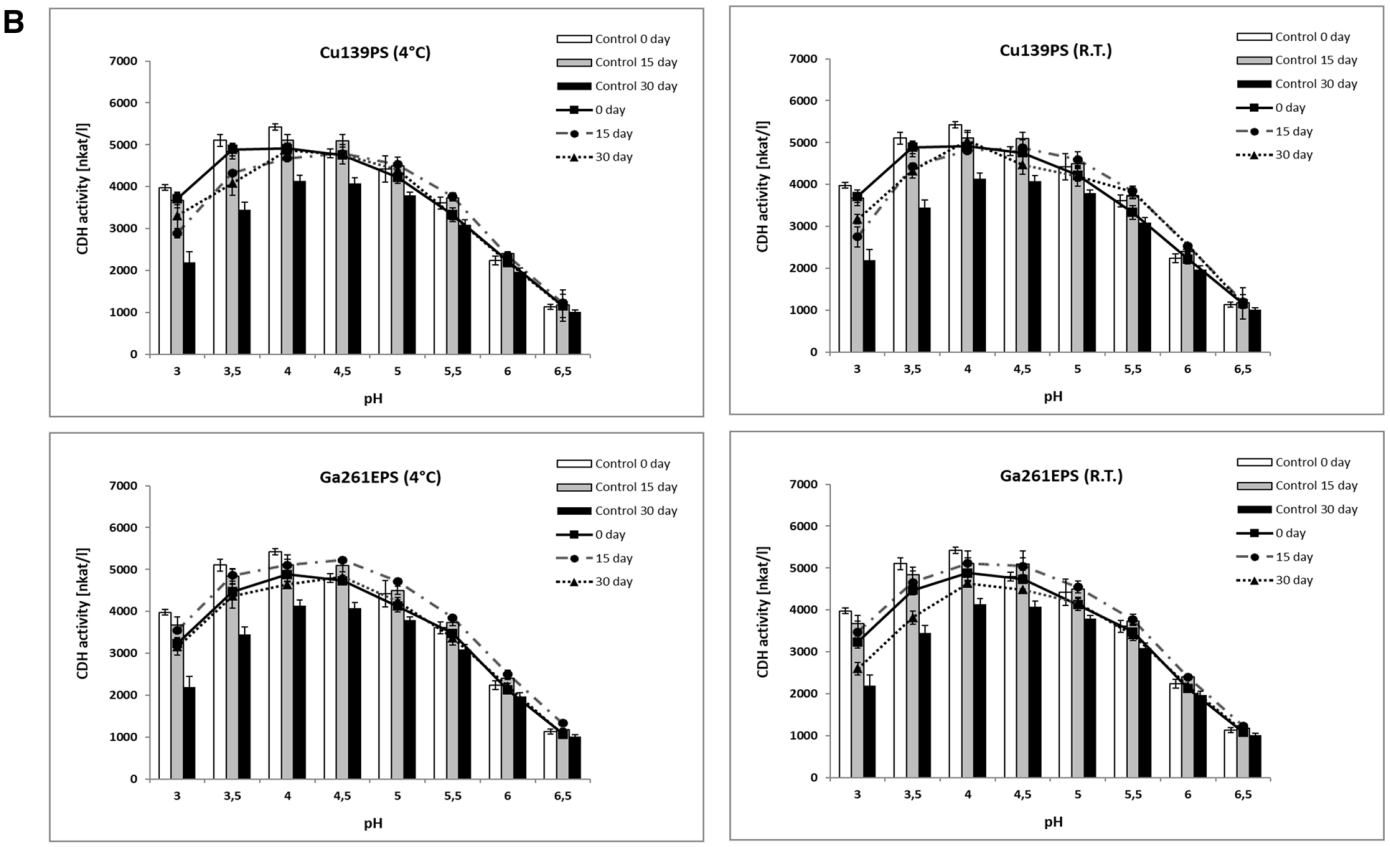
Effect of pH on cellobiose dehydrogenase activity in the presence of polysaccharides. **A** bacterial exopolysaccharides from *R. leguminosarum bv. trifolii* Rt24.2 (Rh24EPS), *B. elkanii* USDA76 (Rh76EPS), *B. japonicum* USDA110 (Rh110EPS), and *S. meliloti* Rm1021 (Rh1021EPS) and **B** fungal *C. unicolor* (Cu139PS) and *G. applanatum* (Ga261EPS) polysaccharides at a temperature of 4 °C and room temperature R.T with DCIP as electron acceptors and time of incubation 0, 15, and 30 days. The experiments were performed in triplicate and the average relative value (*n* = 3) and error bars are shown. Values with different letters are significantly different (*p* ≤ 0.05)

The activity and stability of enzymes largely depend on the primary structure of the protein but the pH ranges in the reaction environment have a considerable influence on the final effects of any biocatalyst. Protein molecules are very sensitive to changes in pH values and exhibit the best functional properties at specific optimal pH. Extreme changes in pH can cause ionization of the functional groups of amino acids and, finally, conformational changes leading to loss of activity (Dill 1990). The use of modifier effectors, such as polysaccharides, at different pH ranges, probably allows charging the protein surface and, consequently, changing the isoelectric constant of the surroundings (Altikatoglu and Basaran 2011), thus stabilizing the protein structure. Frequently, the addition of preservation substances can shift the optimal pH value of the enzymatic catalysis towards acid or alkaline values. The activity of CDH from *P. sanquineus* after incubation in different pH (3.0–6.5) conditions was measured to test the influence of various polysaccharides on the pH stability of CDH. The CDH storage stability in the absence and presence of polysaccharides as additives were studied both at 4 °C and at room temperature to simulate refrigerated and ambient reaction conditions. The results are presented in Fig. 2A and B. It was observed that the optimal pH of the native enzyme catalysis conditions depended evidently on the storage time. After 15 days of the experimental period, the optimum pH shifted from 4.0 to 4.5. Our previous results regarding the biochemical properties of CDH from *P. sanguineus* (FCL 199) have shown that the optimum value pH is 4.5 (Sulej et al. 2013), whereas the pH optimum of CDH from other Basidiomycota is in the range from 4 to 5 (Ludwig et al. 2013). A similar pH range was observed in preparations incubated with polysaccharides, but the addition of Rh110EPS and Cu139PS caused a slight shift in pH to acidic values. Furthermore, all the polysaccharides tested had a stabilizing effect, maintaining higher CDH activity particularly at pH 3.0 after 30 days of incubation in the refrigerated conditions (4 °C), compared to the control enzyme. Changes in the optimal pH value were also observed in studies of the properties of native horseradish peroxidase (Altikatoglu and Basaran 2011) and glucose oxidase (Altikatoglu and Basaran-Elalmis 2012) in the presence of dextran as an additive. Maximum pH was shifted from 4.0 to 5.0 in the presence of dextran as a modification factor (Altikatoglu and Basaran-Elalmis 2012; Altikatoglu and Basaran 2011).

#### Detection of CDH activity using native PAGE electrophoresis

**Fig. 3:**
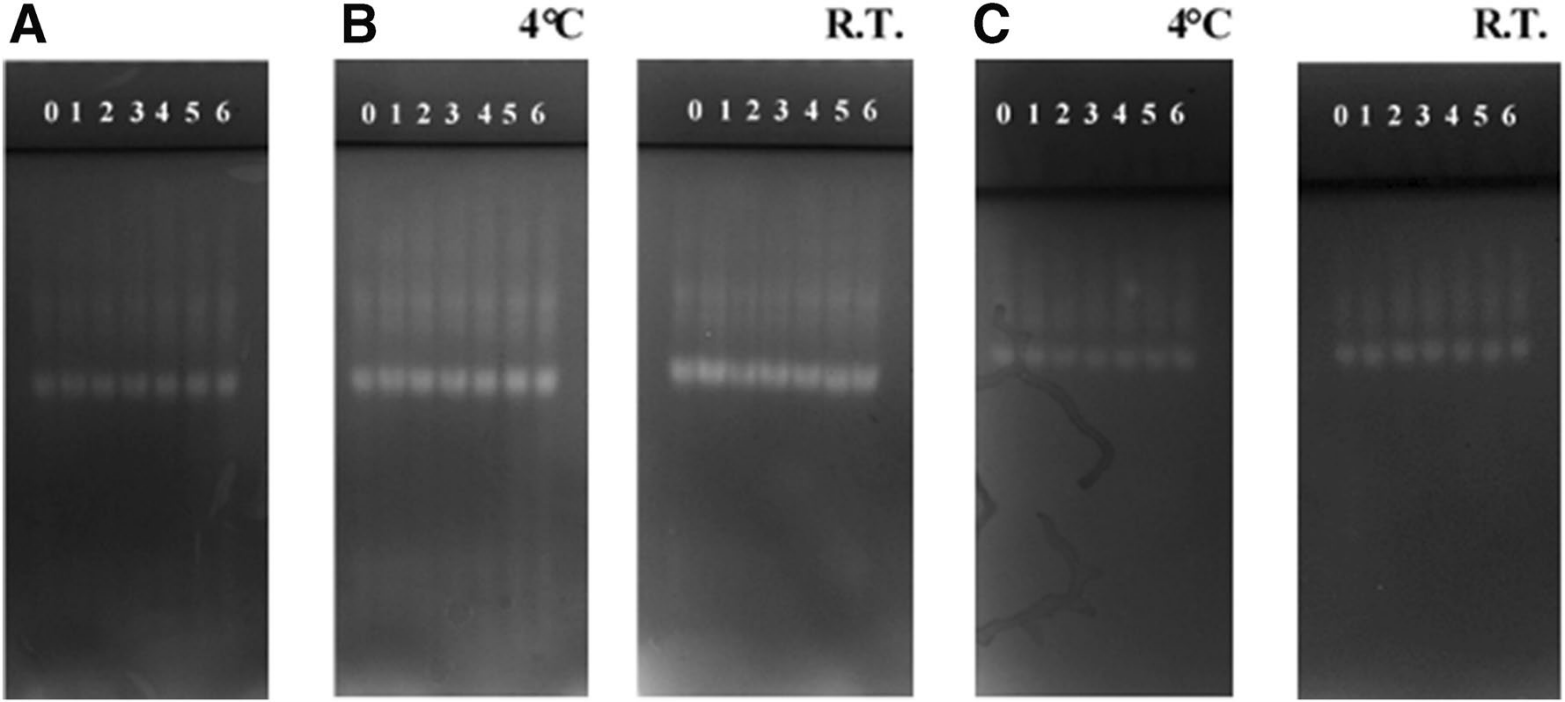
Native PAGE electrophoresis of cellobiose dehydrogenase activities with and without polysaccharides. **A** Control (time of incubation 0), **B** electrophoresis of CDH activities after 15 days of incubation, and **C** electrophoresis of CDH activities after 30 days of incubation. CDH activity without additives (0) and with bacterial exopolysaccharides: Rh24EPS from *R. leguminosarum bv. trifolii* Rt24.2 (1), Rh1021EPS from *S. meliloti* Rm1021 (2), Rh110EPS from *B. japonicum* USDA110 (3), Rh76EPS from *B. elkanii* USDA76 (4) and fungal polysaccharides Cu139PS from *C. unicolor* (5) and Ga261EPS from *G. applanatum* (6)

Native PAGE electrophoresis was conducted for the *P. sanguineus* CDH isolated and purified as described above. The zymographic analysis of the enzyme samples incubated with the polysaccharide showed that the CDH catalytic activity was stable throughout the experimental period. The intensity of the bands corresponding to the enzyme activity was the highest at time 0 and after 15 days of incubation with bacterial and fungal polysaccharides in both investigated temperature ranges. After 30 days, the enzyme activity declined but was still at a level close to the control (without polysaccharide addition). The highest activity of modified CDH was also observed in the case of time 0 and 15-day incubation, in comparison to the control. It should be emphasized that the mobility of the protein in the polyacrylamide gel did not change during the whole experiment (Fig. 3). The analyses confirmed the results of spectrophotometric measurements of CDH activity in the presence of the polysaccharides used and showed their preservation effect on the structural integrity of the enzyme molecules.

#### Kinetic parameters of CDH in the presence of PSs

The kinetic constants of both free and modified cellobiose dehydrogenase were calculated from the Michaelis–Menten curve by varying the concentration of lactose or cellobiose as reaction substrates in optimum conditions (Table 2). Significant changes in the values of catalytic constants, especially the Michaelis–Menten constant (*K*_m_), were observed in the presence of cellobiose as a CDH substrate. At time 0, the value of *K*_m_ of all CDH-PS complexes was slightly higher than that of the control enzyme (0.158 mM). The highest *K*_m_ values were obtained for bacterial polysaccharides Rh76EPS (0.193 mM) and Rh1021EPS (0.187 mM). A considerable decrement of *K*_m_ was observed during the storage at days 15 and 30 both in the control CDH and the CDH-PS complex in the presence of cellobiose as a reaction substrate. The lowest *K*_m_ values were noted for Rh76EPS (0.05 mM), Rh110EPS (0.070 mM), Cu139PS (0.075 mM), and Rh1021EPS (0.079 mM) after 30 days of incubation at room temperature. The decreasing value of *K*_m_ during storage indicates the increasing affinity of the enzyme molecules to cellobiose. This observation may be related to the favorable change in the structural organization of the enzyme induced by the presence of polysaccharides during catalysis. The changes in the value of *K*_m_, i.e. evidence of the increased affinity of the enzyme to the substrate, has also been reported in studies on immobilization of such enzymes as glucose oxidase (Abbasi et al. 2016), amylase, or inulinase (Chapman et al. 2018). A different effect was observed in the presence of lactose as a substrate. The *K*_m_ value during the storage time increased or remained at a constant level. This may indicate a decline in the affinity of the enzyme molecules to the substrate. Many reports have shown a higher *K*_m_ value (lower affinity to the substrate) of enzymes after the immobilization procedure (Jadhav and Singhal 2014; Milovanović et al. 2007). Significant changes were also observed in the *k*_cat_ value in the presence of both the cellobiose and lactose substrates. Storage of the enzyme at room temperature caused an evident decrease in the *k*_cat_ value, which indicated lower efficiency of the biocatalyst. On the other hand, the incubation of the enzyme solution at 4 °C in the presence of Rh110EPS (cellobiose and lactose) and Rh139EPS (lactose) caused an increase in the *k*_cat_ value in comparison to the control samples. An increase in *k*_cat_ values in the presence of Ficoll was earlier reported in studies of the stabilization of epoxide hydrolase by covalent conjugation with polysaccharides (Zou et al. 2018). In a study on fungal laccase activity, Rh110EPS exerted an opposite effect in the same storage condition, i.e. a two-fold decrease in the k_cat_ value compared to the control (Osińska-Jaroszuk et al. 2018).

**Table 2:** Comparison of *K*_m_ and *k*_cat_ for cellobiose dehydrogenase (CDH) in the presence of bacterial and fungal polysaccharides (PSs)

	Day of incubation					
	0		15		30	
	*K*_m_ (mM)	*k*_cat_ (/s)	*K*_m_ (mM)	*k*_cat_ (/s)	*K*_m_ (mM)	*k*_cat_ (/s)
Cellobiose as substrates						
A) Temperature of incubation 4 °C						
CDH without EPS	0.158 ± 0.01	11.73 ± 0.01	0.122 ± 0.02	9.45 ± 0.03	0.124 ± 0.01	9.42 ± 0.02
Rh24EPS	0.179 ± 0.01	13.60 ± 0.01	0.102 ± 0.02	9.55 ± 0.02	0.113 ± 0.03	8.40 ± 0.03
Rh76EPS	0.193 ± 0.02	10.99 ± 0.01	0.186 ± 0.03	10.64 ± 0.02	0.108 ± 0.02	9.00 ± 0.03
Rh110EPS	0.170 ± 0.06	8.56 ± 0.01	0.103 ± 0.01	9.74 ± 0.01	0.123 ± 0.07	12.46 ± 0.06
Rh1021EPS	0.187 ± 0.01	10.26 ± 0.01	0.085 ± 0.01	10.75 ± 0.01	0.091 ± 0.01	10.04 ± 0.02
Cu139PS	0.174 ± 0.03	9.64 ± 0.05	0.091 ± 0.01	9.08 ± 0.01	0.085 ± 0.03	9.91 ± 0.04
Ga261EPS	0.170 ± 0.01	13.76 ± 0.01	0.080 ± 0.01	10.48 ± 0.01	0.125 ± 0.05	11.43 ± 0.06
B) Room temperature R.T						
CDH without EPS	0.158 ± 0.01	11.73 ± 0.01	0.096 ± 0.01	9.75 ± 0.02	0.097 ± 0.02	6.52 ± 0.02
Rh24EPS	0.179 ± 0.01	13.60 ± 0.01	0.109 ± 0.02	8.49 ± 0.02	0.101 ± 0.01	5.82 ± 0.01
Rh76EPS	0.193 ± 0.02	10.99 ± 0.01	0.095 ± 0.03	9.66 ± 0.02	0.05 ± 0.02	6.14 ± 0.03
Rh110EPS	0.170 ± 0.06	8.56 ± 0.01	0.089 ± 0.01	8.99 ± 0.01	0.070 ± 0.07	7.75 ± 0.06
Rh1021EPS	0.187 ± 0.01	10.26 ± 0.01	0.084 ± 0.01	9.69 ± 0.01	0.079 ± 0.01	6.34 ± 0.02
Cu139EPS	0.174 ± 0.05	9.64 ± 0.03	0.108 ± 0.01	9.69 ± 0.02	0.075 ± 0.01	8.59 ± 0.01
Ga261EPS	0.170 ± 0.01	13.76 ± 0.01	0.086 ± 0.02	9.22 ± 0.01	0.087 ± 0.03	6.49 ± 0.02
Lactose as substrates						
A) Temperature of incubation 4 °C						
CDH without EPS	2.903 ± 0.03	14.30 ± 0.02	2.567 ± 0.04	10.23 ± 0.03	3.309 ± 0.06	10.29 ± 0.02
Rh24EPS	3.390 ± 0.05	16.58 ± 0.01	3.015 ± 0.02	10.06 ± 0.02	4.040 ± 0.03	10.20 ± 0.03
Rh76EPS	3.010 ± 0.03	12.97 ± 0.04	2.950 ± 0.03	10.39 ± 0.02	3.661 ± 0.02	10.44 ± 0.03
Rh110EPS	2.648 ± 0.06	9.72 ± 0.04	3.240 ± 0.02	10.88 ± 0.02	3.002 ± 0.07	12.70 ± 0.06
Rh1021EPS	2.824 ± 0.02	12.07 ± 0.03	2.625 ± 0.04	11.27 ± 0.03	3.288 ± 0.04	11.58 ± 0.02
Rh139EPS	3.050 ± 0.03	10.01 ± 0.05	3.546 ± 0.02	10.85 ± 0.03	3.725 ± 0.03	12.57 ± 0.04
Rh261EPS	3.105 ± 0.04	16.96 ± 0.03	2.433 ± 0.03	9.51 ± 0.04	3.060 ± 0.05	10.65 ± 0.06
B) Room temperature R.T						
CDH without EPS	2.903 ± 0.01	14.30 ± 0.01	3.099 ± 0.04	9.63 ± 0.03	4.121 ± 0.01	6.89 ± 0.02
Rh24EPS	3.390 ± 0.01	16.58 ± 0.03	3.913 ± 0.02	8.45 ± 0.02	5.080 ± 0.06	6.34 ± 0.03
Rh76EPS	3.010 ± 0.02	12.97 ± 0.01	3.952 ± 0.03	9.43 ± 0.02	3.684 ± 0.06	9.11 ± 0.03
Rh110EPS	2.648 ± 0.06	9.72 ± 0.01	2.39 ± 0.01	8.49 ± 0.01	3.54 ± 0.07	8.58 ± 0.06
Rh1021EPS	2.824 ± 0.01	12.07 ± 0.01	2.830 ± 0.01	9.56 ± 0.01	3.406 ± 0.01	8.39 ± 0.02
Rh139EPS	3.052 ± 0.03	10.01 ± 0.05	2.971 ± 0.01	9.33 ± 0.01	4.934 ± 0.03	9.75 ± 0.04
Rh261EPS	3.105 ± 0.02	16.96 ± 0.01	2.847 ± 0.01	8.91 ± 0,01	3.446 ± 0.05	7.13 ± 0.06

All results are expressed as mean ± SD from three experiments (*n* = 3). Values within the columns followed by different letters are significantly different (*p* ≤ 0.05)

### Antioxidative properties of *P. sanguineus* CDH in the presence of bacterial and fungal PSs

**Fig. 4:**
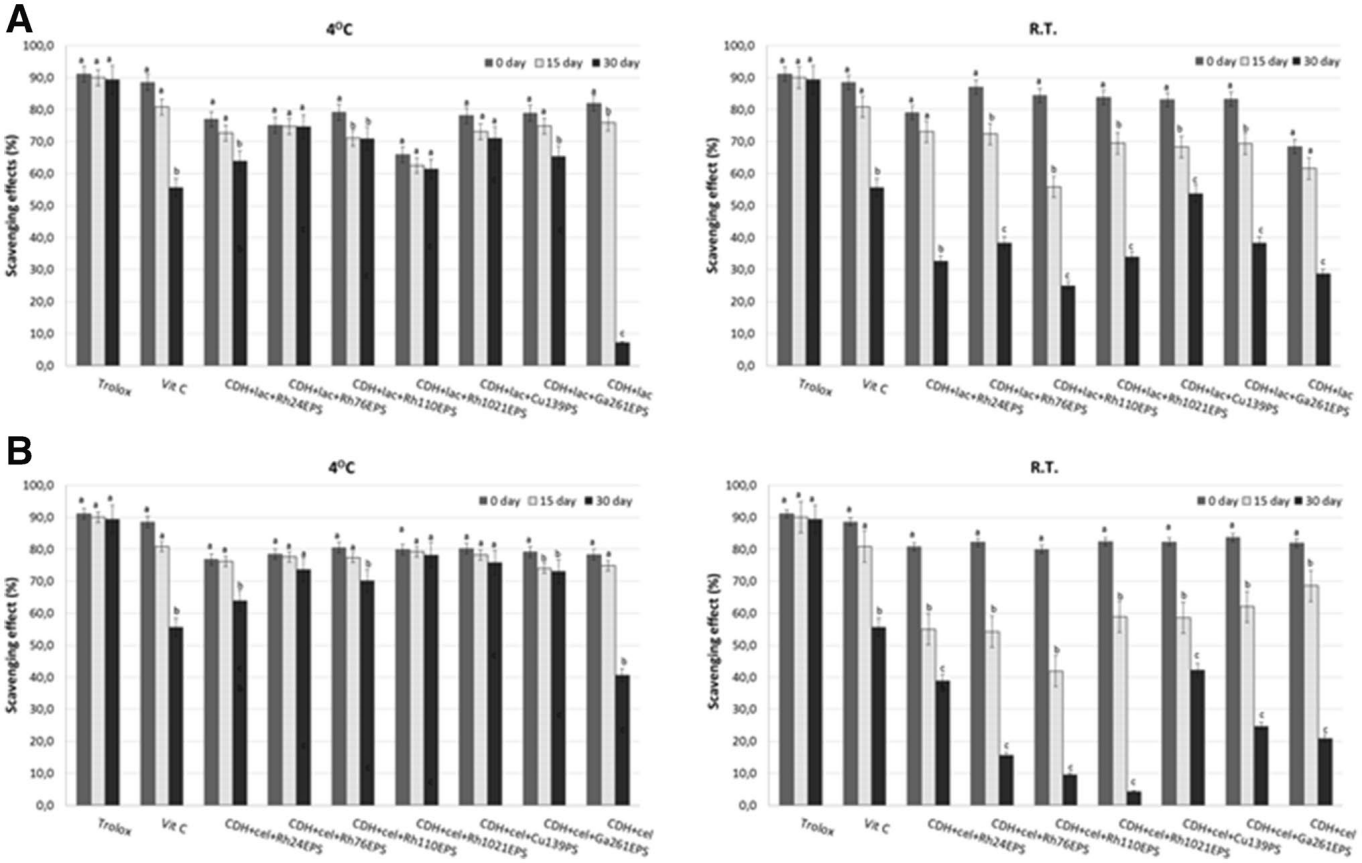
Scavenging effects of cellobiose dehydrogenase in the presence of polysaccharides at a temperature of 4 °C and room temperature R.T and time of incubation 0, 15, and 30 days. The experiments were performed in triplicate and the average relative value (*n* = 3) and error bars are shown. Values with different letters are significantly different (*p* ≤ 0.05). CDH with lactose (**A**) or cellobiose (**B**) as substrates

Fungi have shown the ability to produce many bioactive substances e.g. polysaccharides, polyphenols, vitamins, carotenoids, and minerals with antioxidant properties. In addition, cellobiose dehydrogenases are oxidoreductive enzymes with strong antioxidant properties in the presence of such substrates as cellobiose and lactose. In our previous studies, we proved such properties of fungal CDH from the fungus *P. sanguineus* (Sulej et al. 2013). In the present study, the antioxidative properties of the *P. sanguineus* CDH in the presence of bacterial exopolysaccharides from *R. leguminosarum bv. trifolii* Rt24.2 (Rh24EPS), *B. elkanii* USDA76 (Rh76EPS), *B. japonicum* USDA110 (Rh110EPS), *S. meliloti* Rm1021 (Rh1021EPS), and fungal polysaccharides from *C. unicolor* (Cu139PS) and *G. applanatum* (Ga261EPS) were determined for the first time. Among all the polysaccharides selected for testing, only the polysaccharide isolated from *C. unicolor* had strong scavenging abilities, i.e. approximately 60%, assayed with the DPPH method (Jaszek et al. 2013). The other polysaccharides did not show antioxidant potential (data not shown). The study results indicate that the presence of polysaccharides stabilizes the antioxidant properties of CDH (Fig. 4). After 30 days of storage at 4 °C, a stabilizing effect of all tested polysaccharides (at 61.4–78.1%) on the CDH antioxidative potential was observed, regardless of the type of substrate used (lactose or cellobiose). At room temperature, the best scavenging effect was detected for CDH stabilized with Cu139PS (42.2–53.8%) and Rh24EPS (32.6–38.9%). It is known that the presence of a polysaccharide-protein complex can affect antioxidant properties. Polysaccharides isolated from *Lentinula edodes* and *Schizophyllum commune*, in which the presence of proteins was detected in trace amounts, did not exhibit substantial antioxidative activity. In turn, the polysaccharide-protein complex from *Coriolus versicolor* mycelium with a higher share of proteins has proven to be more effective in antioxidative activity (Liu et al. 1997). In the present study, the use of Cu139PS polysaccharide probably enhanced the antioxidant properties of the enzyme, which resulted from the ability of this polysaccharide to scavenge free radicals. In the case of the other sugar polymers tested, their stabilizing effect on the enzyme may be due to their chelating ability. However, more research is needed to clarify the proper mechanisms underlying the enzyme stabilizing ability of polysaccharides.

### Electrochemical parameters of cellobiose dehydrogenase in the presence of bacterial and fungal PSs

**Fig. 5:**
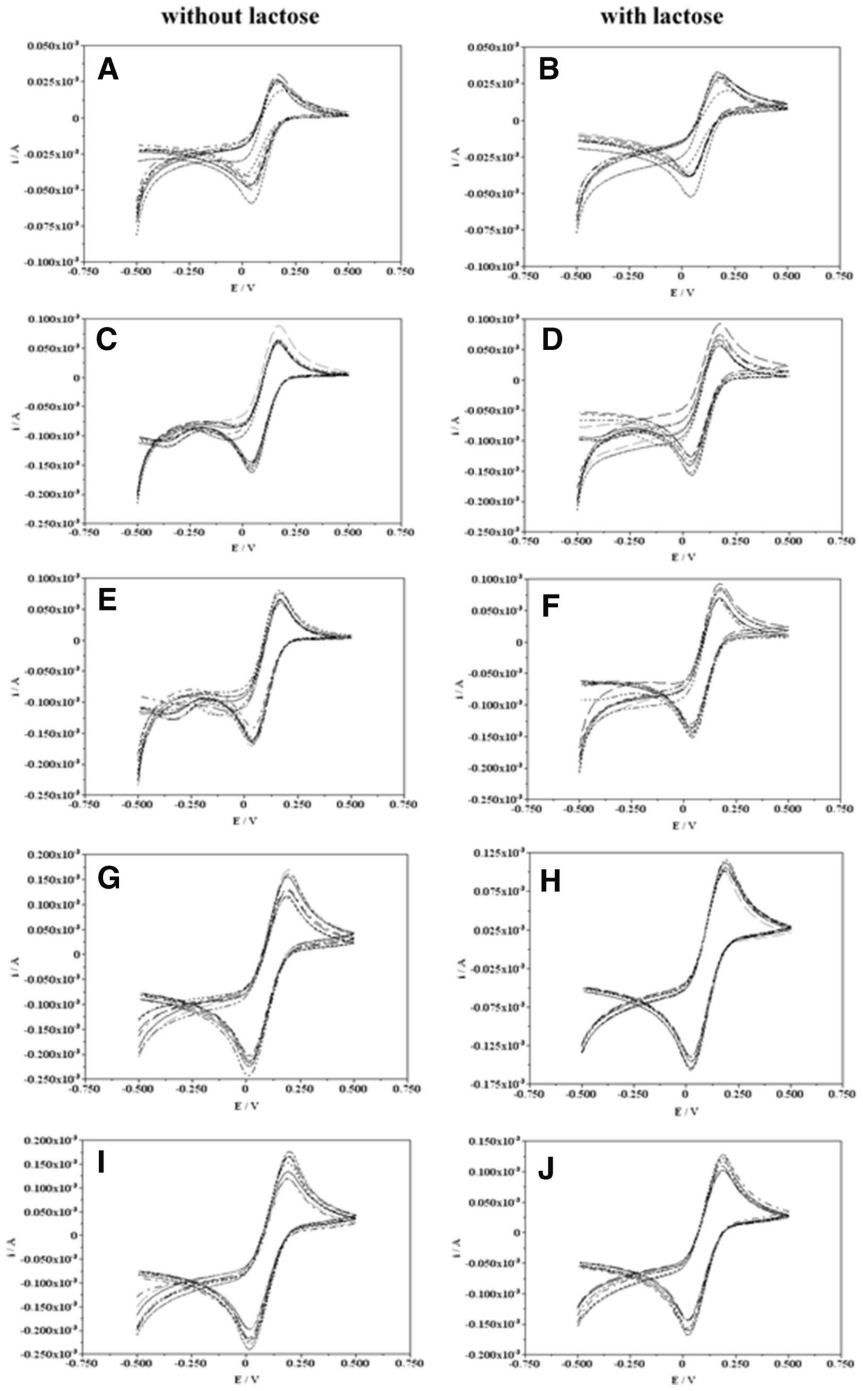
Electrochemical characterization of CDH from *P. sanguineus* strain with polysaccharides (PS). **A**, **B** day 0, **C**, **D** day 15, 4 °C, **E**, **F** day 15, R.T, **G**, **H** day 30, 4 °C, **I**, **J** day 30, R.T. Cu139PS -------, Rh110EPS ………, Rh76EPS --.--.--., Rh1021EPS -------, Rh24EPS --..--..--, Ga261EPS xxxxxxx)

The registered redox wave of modified CDH depends strongly on the time of incubation with polysaccharides and on the type of polysaccharide (Fig. 5). Each enzyme variant generated a well-shaped anodic peak and one cathodic peak both in the presence of the 30 mM lactose substrate and without the substrate. CDH voltametric peaks increased to some extent with the duration of the incubation of the enzyme with the polysaccharide. The highest peak values were recorded after 30 days of incubation at 4 °C. This may be related to changes in the force of interaction between CDH and the electrode caused by the presence of polysaccharides. A comparison of the effect of the polysaccharides tested on the electrochemical properties of CDH demonstrated the largest peak shifts for the fungal polysaccharide obtained from *C. unicolor* (Cu139PS). These results indicate that in all variants of CDH incubation with the tested polysaccharides, both fungal and bacterial, the PSs improved the stability of the enzyme in the electrochemical experimental conditions. This may be related to the effect of polysaccharides on the internal electron transfer in the enzyme molecule. CDH is an extracellular flavohemoenzyme comprising two prosthetic groups, flavin (FAD) and heme (cytochrome b type), located in two separate domains and connected by a flexible linker region that can be cleaved by proteases (Hallberg et al. 2000; Zámocký et al. 2004). The electrochemical properties of CDH are associated with e.g. internal electron transfer between these domains. The rate of internal electron transfer is largely dependent on various factors, such as pH or temperature (Lindgren et al. 2000; Stoica et al. 2005, 2004). Thiols with terminal alcohol groups (hydrophilic/uncharged functional groups) have been proven to locate the CDH molecule in an optimal position in order to obtain the most favorable electronic communication with the electrode (Stoica et al. 2005). The polysaccharide molecules used in this work are probably able to change the electron transfer by improving the electrochemical stability of CDH molecules.

## Conclusions

The results presented in the work demonstrate a novel approach to the stabilization of cellobiose dehydrogenase activity by modifying the enzyme catalytic properties using natural microbial polysaccharides. The experiments revealed that the addition of PSs ensured much better long-term storage stability of CDH in comparison to the control and also modified the catalytic properties of CDH and its stability in a broad range of pH. Given the present results, the proposed new experimental system of modification of the CDH catalytic properties seems to be a promising tool for biotechnological applications in textile, paper, food, and cosmetic industries, bioremediation processes, medicine, and environmental protection. Natural polysaccharides used as stabilizers increasing the electrochemical properties of CDH can also be very promising for the construction of bioelectrochemical devices such as biosensors or biofuel cells.
